# A Methodology to Assess the Accuracy with which Remote Data Characterize a Specific Surface, as a Function of Full Width at Half Maximum (FWHM): Application to Three Italian Coastal Waters

**DOI:** 10.3390/s140101155

**Published:** 2014-01-10

**Authors:** Rosa Maria Cavalli, Mattia Betti, Alessandra Campanelli, Annalisa Di Cicco, Daniela Guglietta, Pierluigi Penna, Viviana Piermattei

**Affiliations:** 1 Italian National Research Council, Institute for Atmospheric Pollution Research, CNR-IIA, Area della Ricerca di Roma1, Via Salaria Km 29,300, Monterotondo (RM) 00016, Italy; 2 Italian National Research Council, Marine Science Institute CNR-ISMAR, Largo Fiera della Pesca, Ancona (AN) 60125, Italy; E-Mails: mattia.betti@an.ismar.cnr.it (M.B.); a.campanelli@an.ismar.cnr.it (A.C.); p.penna@an.ismar.cnr.it (P.P.); 3 Laboratory of Experimental Oceanology and Marine Ecology DECOS, University of Tuscia, Molo Vespucci, Porto di Civitavecchia, Civitavecchia (RM) 00053, Italy; E-Mails: a.dicicco@unitus.it (A.D.C.); v.piermattei@unitus.it (V.P.); 4 Italian National Research Council, Institute of Environmental Geology and Geoengineering, CNR-IGAG, Area della Ricerca di Roma1, Via Salaria Km 29,300, Monterotondo (RM) 00016, Italy; E-Mail: daniela.guglietta@igag.cnr.it

**Keywords:** accuracy, characterization capability, FWHM, *in situ* hyperspectral reflectance, spectral similarity measurements, coastal water reflectance, CHRIS data, Landsat5-TM data, MIVIS data, PRISMA data

## Abstract

This methodology assesses the accuracy with which remote data characterizes a surface, as a function of Full Width at Half Maximum **(**FWHM). The purpose is to identify the best remote data that improves the characterization of a surface, evaluating the number of bands in the spectral range. The first step creates an accurate dataset of remote simulated data, using *in situ* hyperspectral reflectances. The second step evaluates the capability of remote simulated data to characterize this surface. The spectral similarity measurements, which are obtained using classifiers, provide this capability. The third step examines the precision of this capability. The assumption is that *in situ* hyperspectral reflectances are considered the “real” reflectances. They are resized with the same spectral range of the remote data. The spectral similarity measurements which are obtained from “real” resized reflectances, are considered “real” measurements. Therefore, the quantity and magnitude of “errors” (*i.e.*, differences between spectral similarity measurements obtained from “real” resized reflectances and from remote data) provide the accuracy as a function of FWHM. This methodology was applied to evaluate the accuracy with which CHRIS-mode1, CHRIS-mode2, Landsat5-TM, MIVIS and PRISMA data characterize three coastal waters. Their mean values of uncertainty are 1.59%, 3.79%, 7.75%, 3.15% and 1.18%, respectively.

## 1. Introduction

A lot of remote sensors are dedicated to analysing and monitoring a specific surface, therefore the properties of these sensors are developed in order to optimize the characterization of this surface (*i.e.*, spectral range, Full Width at Half Maximum—FWHM, spectral sampling, *etc.*); the Coastal Zone Colour Scanner (CZCS), MEdium Resolution Imaging Spectrometer Instrument (MERIS) and Sea viewing Wide Field-of-view Sensor (SeaWiFS), all of which are devoted to studying the colour of the sea, are probably the best known examples.

The advent of hyperspectral technology seems to overcome this constraint. As a matter of fact, the narrow and contiguous spectral bands obtained with this technique provide detailed information about every individual element in an image and increase the probability of finding a unique characteristic for any given element which better distinguishes it from the other elements in the image [1]. The concept of hyperspectral imaging was formulated by Goetz *et al.* [2] in order to achieve the primary results of the technique of imaging spectrometry in mineral exploration. However, hyperspectral data have been used in a variety of fields [3,4]. For example, Hyperion satellite data have been used to study buried archaeological structures [5], minerals and soil [6,7], oil slick thickness [8], seagrass and the algal blooms [9–11], shallow water bathymetry [12,13], snow and ice [14,15], the urban surface [16–18], vegetation [19,20], volcanic plumes [21] and water column constituents [22–24].

Due to improvements in sensor technology, it is now possible to acquire different kinds of hyperspectral imaging data from airborne and satellite platforms. As a matter of fact, airborne hyperspectral data, which are characterized by using different types of spectrometers, are currently, available (*i.e.*, Airborne Imaging Spectrometer—AISA, Advanced Airborne Hyperspectral Imaging System—AAHIS, Airborne Hyperspectral Scanner—AHS, Airborne Prism Experiment—APEX, Airborne Visible Infrared Imaging Spectrometer—AVIRIS, Compact Airborne Spectrographic Imager—CASI, Environmental Protection System—EPS–H, Digital Airborne Imaging Spectrometer—DAIS, Hyperspectral Digital Imagery Collection Experiment—HYDICE, Hyperspectral Mapper—HyMap, Multispectral Infrared and Visible Imaging Spectrometer—MIVIS, Reflective Optics System Imaging Spectrometer—ROSIS, *etc.*).

In November 2000 the U.S. National Aeronautics and Space Administration (NASA) launched Hyperion, the first civil hyperspectral sensor, aboard the Earth Observation satellite platform (EO-1) [25]. In October 2001 the European Space Agency (ESA) launched the Compact High-Resolution Imaging Spectrometer (CHRIS), a hyperspectral satellite sensor, as part of the Project for the On-Board Autonomy (PROBA) platform system [26].

Moreover, three new hyperspectral satellite missions (*i.e.*, Environmental Mapping and Analysis Program—EnMAP [27], Hyper-spectral Imager Suite—HISUI [28] and PRecursore IperSpettrale of the application mission—PRISMA [29,30]), of the German, Japanese and Italian Space Agencies, respectively, have recently started and the launches of these satellites have been programmed to take place in the near future.

Every step of the processing of the remote data is focused on the improvement of the characterization of areas of interest [31,32]. In fact, the calibration [33,34], the atmospheric [35,36] and the geometric [37] corrections of the remote data are all devoted to improving the remote data accuracy. The correction of sun glint effects is also dedicated to reducing the uncertainties in the characterization of the water body in open sea and in coastal areas [38]. In particular the amount of the uncertainties, which are related to the calibration, can be evaluated to range from 5% to 15% as a function of sensor type [32,34] and the amount of the uncertainties, which are related to the atmospheric correction, can be evaluated at 1% to 2% as a function of spectral bands and surfaces type [36]. Moreover the selection of the methodology, which characterizes and classifies of areas of interest, is performed in order to obtain the best accuracy of the products. In the literature the amount of the uncertainties, which are related to the characterization the optical water parameters of the coastal area obtained by using the radiative transfer theory, can be evaluated at 5% to 20% [23,39,40].

All these remote data processing steps are applied to the identified and the acquired data. Therefore, the accuracy with which remote data characterize a specific surface depends, in the beginning, on the characteristics of the sensor. The proposed FWHM methodology evaluates the accuracy as a function of a spectral characteristic of the remote sensor. This methodology is focused on the evaluation of the number of the bands in the specific spectral range of the remote sensor. This methodology does not compare the capabilities of different remote sensors, because each remote sensor presents specific spectral characteristics (*i.e.*, spectral range, spectral resolution, *etc.*). On the contrary this methodology explores the spectral characteristic of the each remote sensor (*i.e.*, hyperspectral sensor or multispectral sensor, satellite sensor or airborne sensor) in order to lead the identification of the remote data which improve the characterization of a specific surface. In fact, this methodology was developed on one multispectral (*i.e.*, Landasat5 Thematic Mapper—TM) and four hyperspectral (*i.e.*, CHRIS acquired in mode 1 and mode 2, MIVIS and PRISMA) datasets. The Landasat5-TM and two hyperspectral datasets were generated during an integrated campaign; in particular one satellite (*i.e.*, CHRIS mode 2) and one airborne (*i.e.*, MIVIS) hyperspectral data collection were carried out. This integrated multi-hyperspectral campaign was conducted during the summer of 2011 in three coastal areas in southern Italy, jointly with *in situ* water references. Therefore, this methodology was applied to evaluate the accuracy values, with which the CHRIS-acquired in mode 1 and mode 2, the Landsat5-TM, the MIVIS and the PRISMA data characterize the coastal waters of the area close to the Lagoon of Lesina, the Gulf of Manfredonia and the Gulf of Taranto, as a function of FWHM.

Previous research was carried out by Hochberg and Atkinson [41]; that paper presented a method to evaluate the capability of the multispectral and hyperspectral remote sensors in identifying three coastal substrates cover types (*i.e.*, coral, algae, and sand). The authors used the *in situ* spectral reflectance in order to simulate sensor-specific spectral reflectance of five multispectral remote sensors, three real (*i.e.*, Ikonos, Landsat-ETM+—Enhanced Thematic Mapper Plus and Spot High-Resolution Visible—HVR) and two hypothetic (*i.e.*, Proto and Coral Reef Ecosystem Spectro-Photometric Observatory—CRESPO), and two existing hyperspectral (*i.e.*, AAHIS and AVIRIS) remote sensors. These evaluations employed spectral mixing analysis to discriminate pure and mixed spectra [41].

Meanwhile, van der Meer [42] evaluated the effectiveness of the spectral similarity measures on synthetic and real (*i.e.*, AVIRIS) hyperspectral data. Van der Meer [42] used four spectral measures (*i.e.*, Euclidean Distance Measured, Spectral Angle Measured, Spectral Correlation Measure and Spectral Information Divergence) to assess the similarity of spectral measures among three hydrothermal alteration minerals. Then, the author utilized two statistical parameters to evaluate the performance of these four spectral measures.

## 2. Study Area and Data

An integrated multi-hyperspectral campaign was conducted in the summer of 2011 in three coastal areas in southern Italy. In the study of the marine and coastal waters, integrated campaigns (*i.e.*, simultaneous acquisition of the satellite, airborne and *in situ* data) are acutely needed to calibrate and validate remote data, improve the quality of remote data by means of a more accurate atmospheric correction [43,44], calibrate the bio-optical models and validate the results [23,39,40].

### 2.1. Study Area

**Figure 1.**
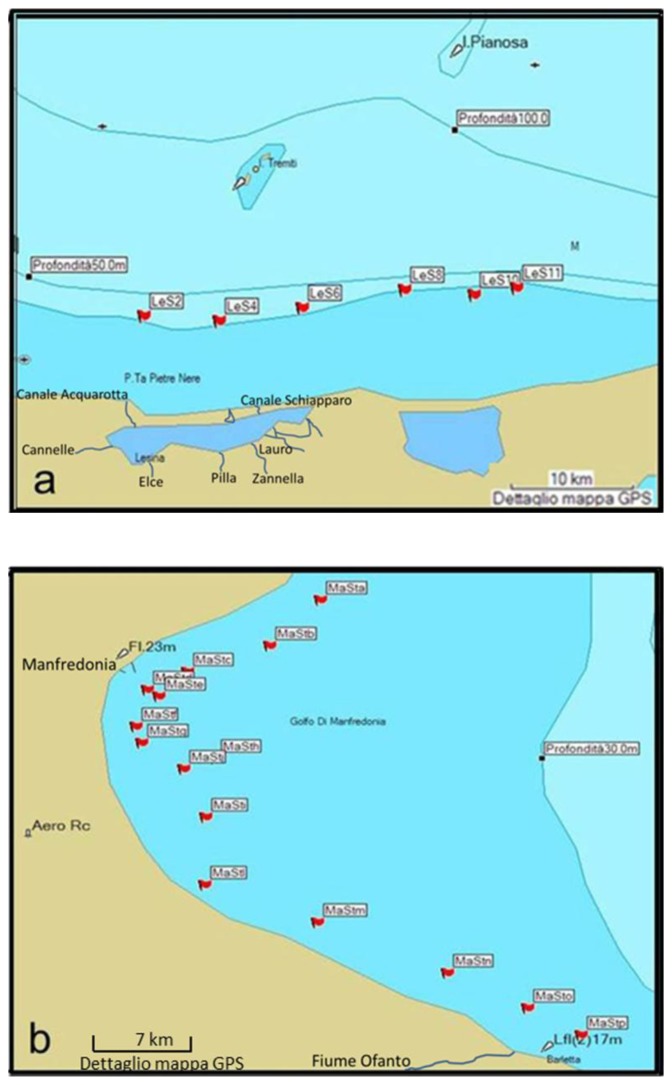

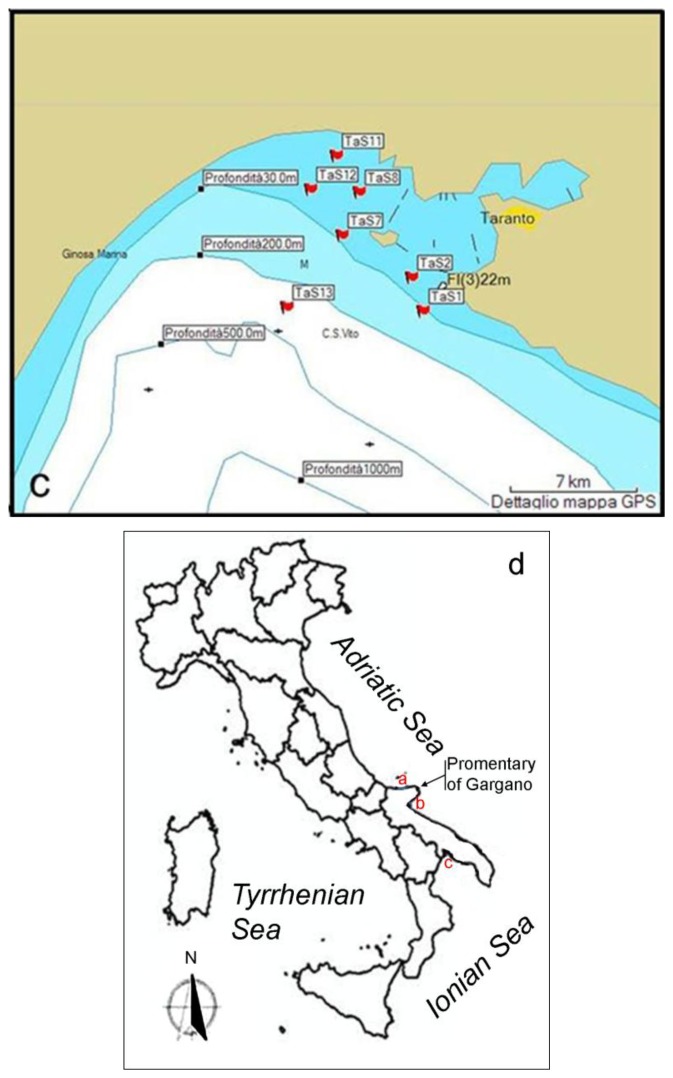
The station locations of the three study areas are shown on the bathymetry maps: (**a**) station names and locations of the coastal waters close to the Lagoon of Lesina; (**b**) station names and locations of the coastal waters of the Gulf of Manfredonia; (**c**) station names and locations of the coastal waters of the Gulf of Taranto; (**d**) location of the coastal waters close to the Lagoon of Lesina, the Gulf of Manfredonia and the Gulf of Taranto, which are highlighted with the letters a, b and c in red, respectively.

The locations of these three surveyed coastal areas are the area close to the Lagoon of Lesina, the Gulf of Manfredonia and the Gulf of Taranto (see Figure 1).

The coastal waters of the area close to the Lagoon of Lesina (red letter a in Figure 1d) are situated along the western part of the southern Adriatic Sea. The Lagoon of Lesina is situated north of the promontory of Gargano (see Figure 1d) and is about 22 km long, with a total area of about 51 km^2^. The lagoon is characterized by shallow water (0.75–1.5 m) and a limited sea-lagoon exchange. The coastal waters of the area close to the Lagoon of Lesina were characterized using six stations. These stations were located in front of the lagoon around the 20 m bathymetric line (see Figure 1a).

The Gulf of Manfredonia (red letter b in Figure 1d) is a large and shallow gulf located in the western part of the southern Adriatic Sea. The Gargano Promontory (see Figure 1d), projecting into the Adriatic Sea, forms the northern border of the gulf and the estuary of the Ofanto river (see Figure 1b) form the southern border. The gulf is named after the town of Manfredonia. The coastal waters of the Gulf of Manfredonia were characterized by 36 stations located in front of the gulf between the 10 m and 15 m bathymetric lines (see Figure 1b). These stations were characterized on different days and the principal positions were monitored several times.

The Gulf of Taranto (red letter c in Figure 1d) is a large square shaped gulf (140 km long by 140 km wide) situated in the Ionian Sea, in southern Italy. The gulf is named after the town of Taranto (see Figure 1c). The coastal waters of the Gulf of Taranto were characterized by 21 stations located at different depths (see Figure 1c). The same positions were monitored several times.

### 2.2. In Situ Measurements

The *in situ* campaign, conducted in these three coastal areas in southern Italy during the summer of 2011, was carried out aboard the ship *Dallaporta* belonging to the Italian National Research Council (CNR). The position of the characterized stations is represented in Figure 1.

In each station water reflectance measurements, bio-physico-chemical parameters and samples of the water column were collected. The choice of the position of each station was based on previous knowledge of the areas and in accordance with the Ocean Optical Protocol [45].

The water samples were collected, at each station, and analyzed in a laboratory in accordance with the protocols laid down by Mueller [46,47]. These analyses were performed to calculate the absorption of the Colored Dissolved Organic Material (CDOM); the absorption of the de-pigmented particles; the absorption of the phytoplankton; the concentration of chlorophyll; the concentration of the Total Suspended Particulate Matter (SPM).

The literature classifies water bodies into two groups (*i.e.*, Case-1 and Case-2 waters) and a lot of intermediary cases; this classifier was originally adopted by Morel and Prieur [48]. The concentration of the phytoplankton is dominant in Case-1 and comparable with that of other particles, while the concentration of the inorganic particles (*i.e.*, SPM) is dominant in Case-2 [48]. The threshold of the concentration of the SPM between Case-1 and Case-2 waters, adopted in accordance with Mueller *et al.* [45], was 0.5 mg/L.

The concentrations of the SPM of the waters of the area close to the Lagoon of Lesina, the Gulf of Manfredonia and the Gulf of Taranto analyzed are more than 0.5 mg/L. Actually, the values of the minimum SPM concentration of the waters of the area close to the Lagoon of Lesina, the Gulf of Manfredonia and the Gulf of Taranto are 1.19, 1.19 and 1.52 mg/L, respectively. Therefore all the waters analysed in this work can be classified as Case-2 or coastal waters in accordance with Mueller *et al.* [45]. Case-2 waters present a high variability, in space and time, of bio-optical parameters [45]. As a matter of fact, each monitored water column was characterized by different bio-chemical and physical parameters, even though the observed water column was monitored in the same position for different days.

#### Water Reflectance Measurements

The coastal water reflectances were recorded using a hyperspectral spectrometer (*i.e.*, FieldSpec FR PRO spectrometer, Analytical Spectral Devices—ASD, Boulder, CO, USA). The hyperspectral spectrometer records in the visible, near infrared and short wave infrared spectral domains. All water reflectances were measured in accordance with the protocols laid down by Mueller [49]. The water reflectances were determined by using the “above water radiance measurements” in accordance with the Ocean Optics Protocols [49]. All “above water radiance measurements” (*i.e.*, Sky Radiance and Water Surface Radiance in air) were acquired using a hyperspectral spectrometer with a field of view of 3°. The radiances were measured from the deck of the ship by using a definite acquisition geometry [50–52]. All “above water radiance measurements” were collected using method 2, denominated “uncalibrated radiance and reflectance plaque measurements” in Mueller *et al.* [49].

Each water column of each station located in the coastal waters of the area close to the Lagoon of Lesina, the Gulf of Manfredonia and the Gulf of Taranto was characterized with at least 10 series of “above water radiance measurements”.

### 2.3. Multi-Hyperspectral Remote Sensing Data

These *in situ* hyperspectral data were simultaneously recorded with the Landsat5-TM, the CHRIS and the MIVIS data on the Gulf of the Manfredonia and with the CHRIS data on the Gulf of the Taranto.

The satellite multispectral data were recorded by the Thematic Mapper (TM) sensor on board the Landsat5 satellite. One Landasat5-TM acquisition was carried out on the coastal area close to the Lagoon of Lesina and the Gulf of Manfredonia on August 9, 2011 and one Landasat5-TM acquisition was carried out on the Gulf of Taranto on August 2, 2011 (data available from USGS archive).

The satellite hyperspectral data were recorded by the CHRIS sensor on board the Project for On-Board Autonomy (PROBA) satellite [26,53]. The satellite acquisitions were obtained through the European Space Agency Category 01 (ESA-CAT-01) project (ID7977) proposed by the Institute of Atmospheric Pollution Research. This project aims to cooperate in the assessment of a bio-optical model in the coastal waters of the Ionian and the Adriatic seas using hyperspectral data. In this framework, eight CHRIS acquisitions were carried out during the summer of 2011 (*i.e.*, three over the Gulf of Manfredonia and five over the Gulf of Taranto). The satellite configuration utilized during this integrated campaign, was mode 2. This configuration is called also “water bands”. In this study two CHRIS imaging configurations were evaluated, mode 1 and mode 2, in order to identify the best configuration for characterizing these coastal waters. The CHRIS data acquired in mode 1 records 62 bands and this configuration includes the bands of other configurations. The CHRIS data acquired in mode 2 records 18 bands and this configuration is devoted to water study. Actually, the wavelength allocations of the CHRIS data acquired in mode 1 are the nominal values in accordance with the CHRIS data format [53], whereas the spectral characteristics of the CHRIS data acquired in mode 2 are the actual wavelengths for the operating temperature, detailed within their HDF files, carried out during the summer of 2011.

The airborne hyperspectral data were acquired by the MIVIS sensor on board a CASA C212 aircraft [54]. The spectral characteristics which were utilized in this paper are certificated by the spectral calibration which was performed in May 2011 by the ArgonST Imaging Group. The MIVIS data were acquired over the Gulf of Manfredonia on August 9, 2011. This acquisition was performed at an absolute altitude of 1,500 m a.s.l.: the same as the pixel ground resolution which is of about 3 m. The MIVIS survey included three flights that were oriented NW/SE parallel to one another.

## 3. Methodology

**Figure 2.**
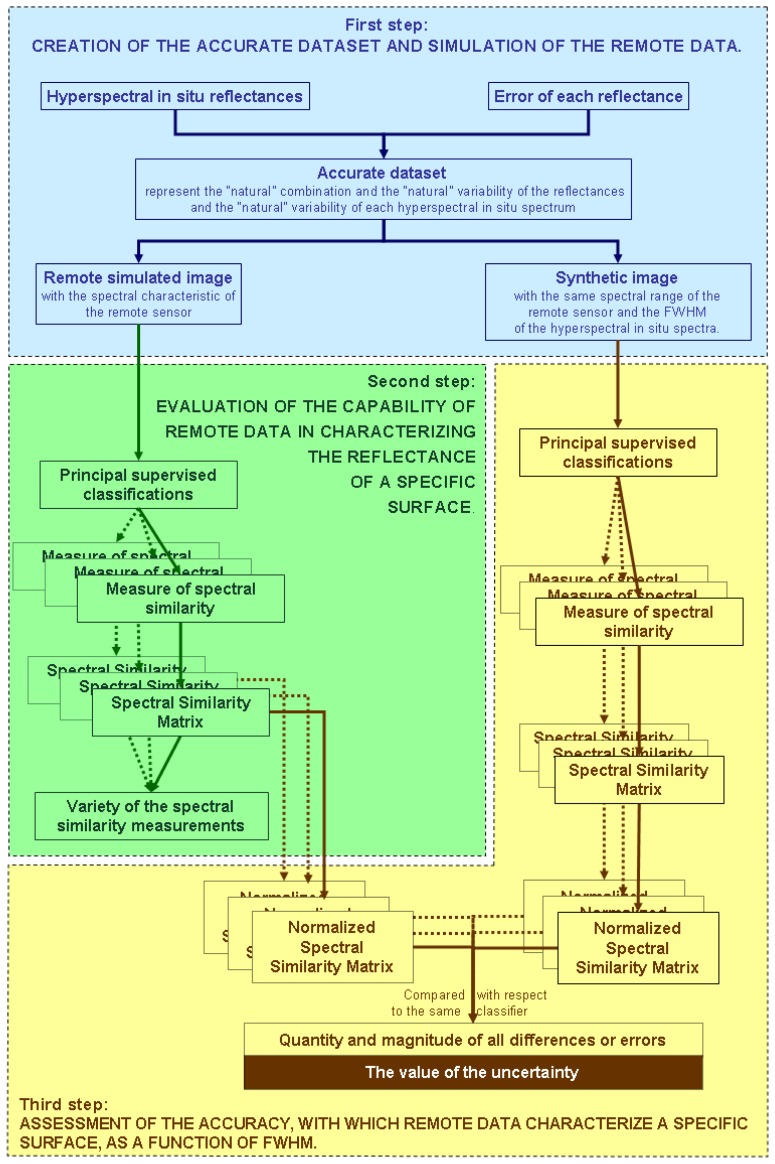
Flow chart of the proposed methodology.

All steps of the processing of the acquired remote data are devoted to optimizing the characterization of a specific surface [31–38]. In the beginning, the accuracy with which remote data characterizes a specific surface depends on the identification of the sensor. This paper proposes a methodology leading to the identification of the remote data, which improves the spectral characterization of the studied surfaces. This methodology assesses the accuracy value with which remote data characterize a specific surface as a function of a spectral characteristic of the sensor—the FWHM. The methodology evaluates the accuracy value of the remote data decreasing their FWHM, therefore, increasing their number of the bands, within the specific spectral regions of the sensor. The proposed methodology is divided into three steps. The flow chart of this methodology is depicted in Figure 2.

### 3.1. Creation of the Accurate Dataset and Simulation of the Remote Data

The first step is performed to create a dataset which accurately describes a specific surface. This dataset is used to better simulate the remote data. In this methodology the hyperspectral reflectances, measured *in situ*, are considered as “real” surface reflectances. In fact, these measurements more accurately characterize the reflectances of the surface with respect to the other remote sensors. Therefore these reflectances are used to create the dataset.

A specific surface is characterized by several different reflectances. This set of reflectances determines their “natural” variability and their “natural” combination. Moreover, the “natural” variability of each reflectance is described by the mean and standard deviation values of the measurements. The range of the mean and the standard deviation values is separated into many steps which have an equal period. All these spectra are used to create an equal number of images. These images have equal dimensions: the number of the lines and the number of the rows of these images are not important, but the number of the bands must be equal to the *in situ* hyperspectral reflectances. These images are jointed to create a mosaic image, which shows the “natural” variability of this *in situ* hyperspectral reflectance.

The “natural” combinations of the specific surface are described by connecting each mosaic image of every *in situ* hyperspectral reflectance. This connection creates a greater mosaic, which accurately describes a specific surface in order to better simulate the remote data.

Therefore, this greater mosaic is spectrally resized as a function of the spectral characteristics of the remote sensor, in order to simulate the remote data avoiding the atmospheric effects and the noise of the remote sensor. Furthermore, a corresponding synthetic image is created. This synthetic data is characterized by the same spectral range of the remote sensor, but different spectral resolution and different number of the spectral bands with respect to the remote simulated data. Actually, the spectral resolution is equal to the FWHM of the *in situ* hyperspectral reflectances and the number of the spectral bands is a function of this FWHM.

### 3.2. Evaluation of the Capability of Remote Data in Characterizing the Reflectance of a Specific Surface

The step for evaluating the capability of remote data in characterizing the reflectance of a specific surface is based on their ability in distinguishing one spectrum from another of the specific surface, in other words, on their ability in measuring the spectral similarity between one spectrum and another. The spectral similarity measurements are achieved by the principal supervised classifiers [55]. The classifiers used in this methodology are Binary Encoding (BE), Maximum Likelihood (ML), Mahalanobis Distance (MaD), Minimum Distance (MiD), Parallelepiped (Pa), Spectral Angle Mapper (SAM) and Spectral Information Divergence (SID). These principal supervised classifiers are performed on the data. The results of the supervised classifiers are two: a thematic map and rule images. This methodology is focused on the analysis of the rule images, which are obtained by these classifiers, because these images represent the measurements of spectral similarity. Each pixel of these images is a spectral similarity measurement between the spectrum of the corresponding pixel of the input data and the spectrum of each user-defined training class. The number of rule images is the same as those of user-defined training classes.

In this methodology a specific surface is characterized by several different reflectances, therefore each spectral similarity measurement should show different magnitude. The number of the measurements which show different magnitude from the others is called the variety of the spectral similarity measurements. However the variety of these measurements is established by the algorithm characteristics, by the spectral characteristics of the input data and by the study area characteristics. The highest variety of these measurements corresponds to the best capability in characterizing several different reflectances: the variety of these measurements is used to assess the capability of the different classifiers, when are applied to the same data; the variety of these measurements is used to assess the capability of the different data, when are classified using the same classifier.

All these classifiers use different algorithms for clustering each pixel into classes and for measuring the spectral similarity between one spectrum and another. The total number of the measurements which show different magnitude from the others is called the total variety. Together these values optimise the characterization, because it takes into consideration every algorithm which is used to measure the spectral similarity and, so, it takes into consideration every aspect of the spectral features independently from the spectral characteristic of the specific surface. Consequently, the total variety optimizes the evaluation of the capacity of the remote data in characterizing a specific surface.

The total variety, divided by the number of the algorithms and by the number of the reflectances, which characterize a specific surface, is the value of the capability of the remote data in characterizing a specific surface.

### 3.3. Assessment of the Accuracy with Which Remote Data Characterize a Specific Surface, as a Function of the FWHM

In the proposed methodology, the third step assesses the accuracy value with which remote data characterize a specific surface, as a function of FWHM. This step is based on the assumption that the *in situ* hyperspectral reflectances more accurately represent the “real” surface reflectances with respect to the other remote sensors. Therefore, the spectral similarity measurements which are performed on these reflectances, are more accurate measurements than the measurements obtained by other remote sensors. These measurements are considered as the “real” spectral similarity measurements. Consequently, the assessment of this accuracy is performed by comparing the spectral similarity measurements of the remote data with respect to the “real” spectral similarity measurements of the corresponding synthetic data. This data is characterized by the same spectral range of the remote data, but the FWHM is equal to the FWHM of the *in situ* hyperspectral reflectances.

The principal supervised classifiers are also executed on the corresponding synthetic data. The spectral similarity values are organized into symmetric matrices, called Spectral Similarity Matrices. These matrices, together with the Spectral Similarity Matrices obtained from the remote data, are normalized from 100 to 0. The normalized Spectral Similarity Matrices of the remote data are compared with respect to the normalized Spectral Similarity Matrices of the corresponding synthetic image. The comparison is performed by counting their differences.

These differences are considered as “characterization errors”, because the *in situ* hyperspectral reflectances are considered as “real” reflectances and the corresponding synthetic image is a spectrally resized version of the *in situ* hyperspectral reflectances, so the spectral similarity measurements, which are obtained from the corresponding synthetic data, are considered as “real” spectral similarity measurements.

These errors are examined for each pair of normalized Spectral Similarity Matrices which are obtained by the same classifier. These errors obtained by all classifiers are summed. All these errors are taken into consideration together, because the overall results improve the description and characterization of a specific surface. The accuracy with which remote data characterizes a specific surface as a function of FWHM is provided by the mean values of the quantity and the magnitude of errors (*i.e.*, differences between spectral similarity measurements which are obtained using “real” resized reflectances and remote simulated data) which are obtained by all performed classifiers.

The evaluation of the quantity of all these differences is performed by summing all the numbers of all the differences. The value is divided by the total interactions among the spectra and by the number of the classifiers, which were performed. This ratio provides the mean percentage of the quantity of all differences with respect to the total interactions among the spectra. Therefore, the mean percentage of the quantity of all differences provides the value of the error quantities.

The assessment of the magnitude of all these errors is performed by summing the magnitude (*i.e.*, absolute value) of all differences. This value of the magnitude is divided by the total interactions among the spectra, by the period, which is used to normalize the Spectral Similarity Matrices, and by the number of the classifiers, which were performed. Therefore, this ratio (*i.e.*, the mean magnitude of all errors which are obtained by all performed classifiers) provides the value of error magnitudes.

In conclusion, the error quantity multiplied by the error magnitude provides the value of the uncertainty, with which remote data characterize a specific surface, as a function of FWHM. This value is considered as a good measurement of the accuracy, because it provides a full description of the quantity and the magnitude of all errors. Table 1 summarizes the products of these three steps and the values which represent these products.

## 4. Results and Discussion

### 4.1. Creation of the Accurate Dataset and Simulation of the Remote Data

The first step was performed to create three datasets which accurately describe the coastal water reflectances of the area close to the Lagoon of Lesina, the Gulf of Manfredonia and the Gulf of Taranto. These coastal reflectances, achieved using the FieldSpec FR PRO spectrometer, are approximated to zero after 860 nm of wavelength. Therefore, this step was only applied to the first 851 bands of this hyperspectral instrument. These bands correspond to the wavelengths from 350 nm to 1,200 nm. This spectral range was selected to obtain the same spectral limits of the remote data which were analyzed. The FWHMs of these hyperspectral reflectances were resampled from 3 mm to 1 nm, in the spectral range from 350 nm to 1,000 nm, and from 10 nm to 1 nm, in the spectral range from 980 nm to 1,200 nm. This spectral resampling was also selected to obtain the same spectral limits of the remote data which were analyzed.

These coastal waters of the area close to the Lagoon of Lesina, the Gulf of Manfredonia and the Gulf of Taranto were characterized by 6, 36 and 21 *in situ* hyperspectral reflectances, respectively. Each *in situ* hyperspectral reflectance characterized a water column having different bio-chemical and physical characteristics one from the other. These three coastal water bodies were separately elaborated to determine the “natural” combination and the “natural” variability of each one, therefore these three coastal water reflectance datasets are considered three different surfaces.

Each water reflectance was described by mean and standard deviation values of the *in situ* hyperspectral reflectances in order to determine the “natural” variability of each reflectance. This range was divided into five steps. These five spectra were used to create five images. Each image is characterized by the same dimensions (*i.e.*, 100 × 20 pixels × 851 bands). Therefore six, 36 and 21 mosaics, of 100 × 100 pixels × 851 bands, were separately composed to describe the coastal waters of the area close the Lagoon of Lesina, the Gulf of Manfredonia and the Gulf of Taranto, respectively. These datasets were organized into three greater mosaics of six, 36 and 21 mosaics which symbolized these coastal water reflectances. This step was only applied to the visible and near infrared portions of the electromagnetic spectrum, because the *in situ* hyperspectral spectra are approximately zero after 860 nm of wavelength. Therefore, these three greater mosaics were spectrally resampled to simulate the data of the CHRIS sensor acquired in mode 1 and mode 2, the first four bands of the Landsat5-TM sensor and the first spectrometer of the MIVIS and of the PRISMA sensors. These data are called here for convenience only the CHRIS mode 1, the CHRIS mode 2, the Landsat5-TM, the MIVIS and the PRISMA data.

Five corresponding synthetic images were created for these five remote data. In Table 2 the spectral characteristics of each data pair (*i.e.*, the remote data and the corresponding synthetic data) are summarized. Therefore, 15 pairs of data were created to simulated these five remote datasets which were recorded for these three coastal waters.

### 4.2. Evaluation of the Capability of the Remote Data in Characterizing the Reflectance of the Three Italian Coastal Waters

In this application the capability of these five remote data in characterizing the reflectance of the three coastal waters were evaluated. The principal supervised classifiers (*i.e.*, the BE, the ML, the MaD, the MiD, the Pa, the SAM and the SID classifiers) were carried out on these five remote images. Each one of these three coastal areas was represented in five remote data, for a total of 15 images.

These classifiers were clustered into six, 36 and 21 classes, which correspond to the six, 36 and 21 water reflectances of the area close to the Lagoon of Lesina, the Gulf of Manfredonia and the Gulf of Taranto, respectively. These user-defined training classes were identified on the images. These regions of interest included the “natural” variability of each reflectance for each user-defined training class. Moreover, these regions of interest were used, also, to extract by the rule images the spectral similarity measurements.

All spectral similarity measurements were organized into seven Spectral Similarity Matrices corresponding to the seven supervised classifiers. Table 3 shows the seven Spectral Similarity Matrices of the PRISMA data which simulated the coastal waters of the area close to the Lagoon of Lesina. Therefore, a Spectral Similarity Matrix was created for each algorithm (*i.e.*, seven algorithms) on each remote dataset (*i.e.*, five remote datasets) and on each coastal site (*i.e.*, three coastal areas) for a total of 105 Spectral Similarity Matrices.

The varieties of the spectral similarity measurements (*i.e.*, the number of the measurements which have different magnitude from the others) were extracted by each Spectral Similarity Matrix. For example the variety, obtained by BE classifier which was performed on the PRISMA data of the area close to Lagoon of Lesina, is equal to 5 (see Table 3).

In Table 4 the varieties of the spectral similarity measurements obtained by each classifier (*i.e.*, the BE, the ML, the MaD, the MiD, the Pa, the SAM and the SID classifiers) for each site are shown. These varieties were obtained on the CHRIS mode 1 and mode 2, the Landsat5-TM, the MIVIS, the PRISMA and the ASD, resized from 400 to 1,000 nm, data. In the last two columns of the Table 4 the total varieties of the spectral similarity measurements and the capabilities are shown.

The capabilities show the highest values when all classifiers were applied to the PRISMA data, and the second values when all classifiers were applied to the CHRIS mode 1 data. The third values of the capabilities, which were obtained by all classifiers, were applied to the CHRIS mode 2 data, and the fourth values were obtained by all classifiers were applied to the MIVIS data. In the end, the capabilities show the last values when all classifiers were applied to the Landasat5 TM data.

In conclusion, the ranking of these values shows that the best capability of these five remote datasets in characterizing these coastal waters is provided by the PRISMA data, which is followed, in ascending order, by the CHRIS mode 1 data, by the CHRIS mode 2 data, by the MIVIS data and by the Landasat5 TM data.

The total varieties and capabilities of the PRISMA data show the slight differences with respect to the total varieties and the capabilities of the CHRIS mode 1 data. In the same way, the total varieties and the capabilities of the MIVIS data show the slight differences with respect to the total varieties and the capabilities of the CHRIS mode 2 data. As a matter of fact, the number of the bands and the FWHM of the first spectrometer of the PRISMA sensor are similar to the number of the bands and the mean value of the FWHM of the CHRIS mode 1 data. In the same way, the number of the bands and the FWHM of the first spectrometer of the MIVIS sensor are similar to the number of the bands and the mean value of the FWHM of the CHRIS mode 2 data.

Moreover the varieties of the spectral similarity measurements of the BE, the ML, the MaD, the MiD, the Pa, the SAM and the SID classifiers are compared to assess the performance in measuring the spectral similarity between each spectrum of these three coastal waters (see Table 4). This evaluation shows that the highest value of the variety which were obtained for the coastal waters of the area close to the Lesina Lagoon is 17; this value was obtained by the SAM classifier when it was applied to the CHRIS mode 1 and the PRISMA data; the highest value of the variety which were obtained for the coastal waters of the Gulf of Manfredonia is 93; this value was obtained by the MiD classifier when it was applied to the PRISMA data; the highest value of the variety which were obtained for the coastal waters of the Gulf of Taranto is 78; this value was obtained by the SAM classifier when it was applied to the PRISMA data.

The capability values cover the range from 1.05 to 2.35. These low capability values could be due to the algorithm characteristics, to the spectral characteristics of the input data and to the study area characteristics. For the first assumption, the literature has highlighted that the application of models based on the radiative transfer theory, to assess optical water parameters of the coastal area [23,39,40], improves the quality of the results with respect to the other applications. For the second assumption, these low values cannot be due to the spectral characteristics of the input data, because the capabilities of hyperspectral *in situ* data are low too (see Table 5). For the third assumption, these three spectra datasets used to develop this methodology, gave slight differences among them. Actually, the mean values of the spectral similarity measurements, which were obtained by the MiD classifier (the MiD and the SAM classifiers are the highest variety) when it was applied to the water reflectances of the area close to the Lagoon of Lesina, the Gulf of Manfredonia and the Gulf of Taranto, are equal to 0.011, 0.036 and 0.009, respectively. The measurement of the spectral similarity, which is calculated by the MiD classifier between the same spectrum, is equal to 0. The mean values of the spectral similarity measurements obtained by the SAM classifier, when it was applied to the water reflectances of the area close to the Lagoon of Lesina, the Gulf of Manfredonia and the Gulf of Taranto, are equal to 0.078, 0.171 and 0.116, respectively. The spectral similarity measurement, which is calculated by the SAM classifier between the same spectrum, is equal to 0. In conclusion, the low values of the capability of these remote data in characterizing the reflectance of these coastal waters are due to the algorithm characteristics and to the slight differences among these three spectra datasets.

### 4.3. Assessment of the Accuracy with Which Remote Data Characterize the Three Italian Coastal Waters, as a Function of the FWHM

This last proposed step was performed in order to evaluate the accuracy values with which these five remote simulated datasets characterize these three coastal waters, as a function of FWHM. This evaluation was performed by comparing the normalized Spectral Similarity Matrices of each remote dataset with respect to the normalized Spectral Similarity Matrices of each corresponding synthetic dataset. Actually, the comparison was performed on the Spectral Similarity Matrices, which were obtained by the same classifier, and was repeated for each classifier.

Therefore, the BE, the ML, the MaD, the MiD, the Pa, the SAM and the SID classifiers were also executed on all corresponding synthetic images (*i.e.*, five remote simulated datasets for each coastal site were created for a total of 15 synthetic images). The spectral similarity measurements which were obtained with these classifiers, were organized into 105 Spectral Similarity Matrices. The Spectral Similarity Matrices of the remote data and the Spectral Similarity Matrices of the corresponding synthetic data were normalized, and thus the comparison between these two normalized Spectral Similarity Matrices, which were obtained by the same classifier, was performed.

The comparison was evaluated by counting the error quantities and the error magnitudes. Actually, the evaluation of the error quantities was performed by counting, before, the total numbers of the all errors and, then, the mean percentage of the quantity of errors, which are obtained by all performed classifiers. The evaluation of the error magnitudes was performed by counting, before, the total magnitude (*i.e.*, absolute value) of all errors and, then, the mean magnitude of errors, which are obtained by all performed classifiers. Therefore the values of the error quantities and the error magnitudes provide the values of the uncertainty. Table 5 summarizes the results.

The PRISMA data shows the smallest values of the uncertainty with respect to the other remote data in characterizing the coastal waters of the area close to the Lagoon of Lesina, the Gulf of Manfredonia and the Gulf of Taranto. The values of the uncertainty of the PRISMA data are followed, in ascending order, by the CHRIS mode 1, the MIVIS, the CHRIS mode 2 and the Landsat5-TM data. Moreover, the PRISMA data shows the smallest error quantities and the smallest error magnitudes with respect to the other remote data in characterizing the coastal waters of the area close to the Lagoon of Lesina, the Gulf of Manfredonia and the Gulf of Taranto. The error quantities and the error magnitudes of the PRISMA data are followed, in ascending order, by the CHRIS mode 1 data, by the MIVIS data, by the CHRIS mode 2 data and by the Landsat5-TM data.

It should be noted that the value of the uncertainty and the error magnitudes which were calculated for the coastal waters of the Gulf of Taranto with the CHRIS mode 2 data (*i.e.*, 1.63% and 0.46, respectively, see Table 5) are smaller than the value of the uncertainty and the error magnitudes which were calculated for the same coastal waters, using the MIVIS data (*i.e.*, 2.16% and 0.63, respectively, see Table 5).

**Figure 3.**
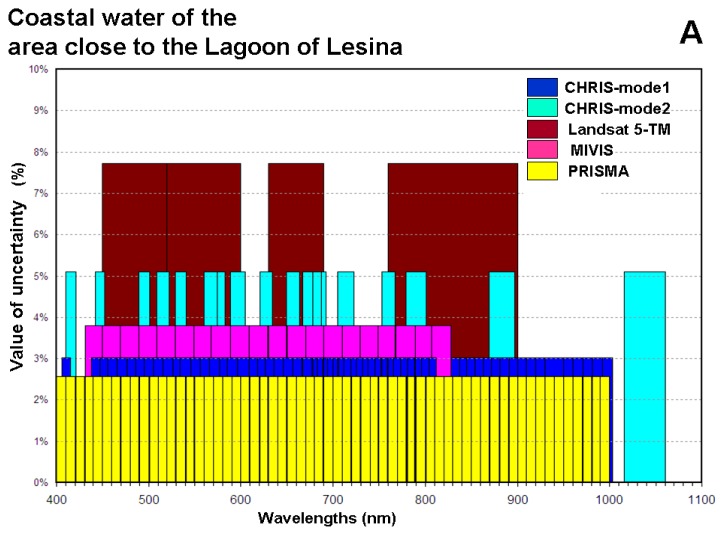

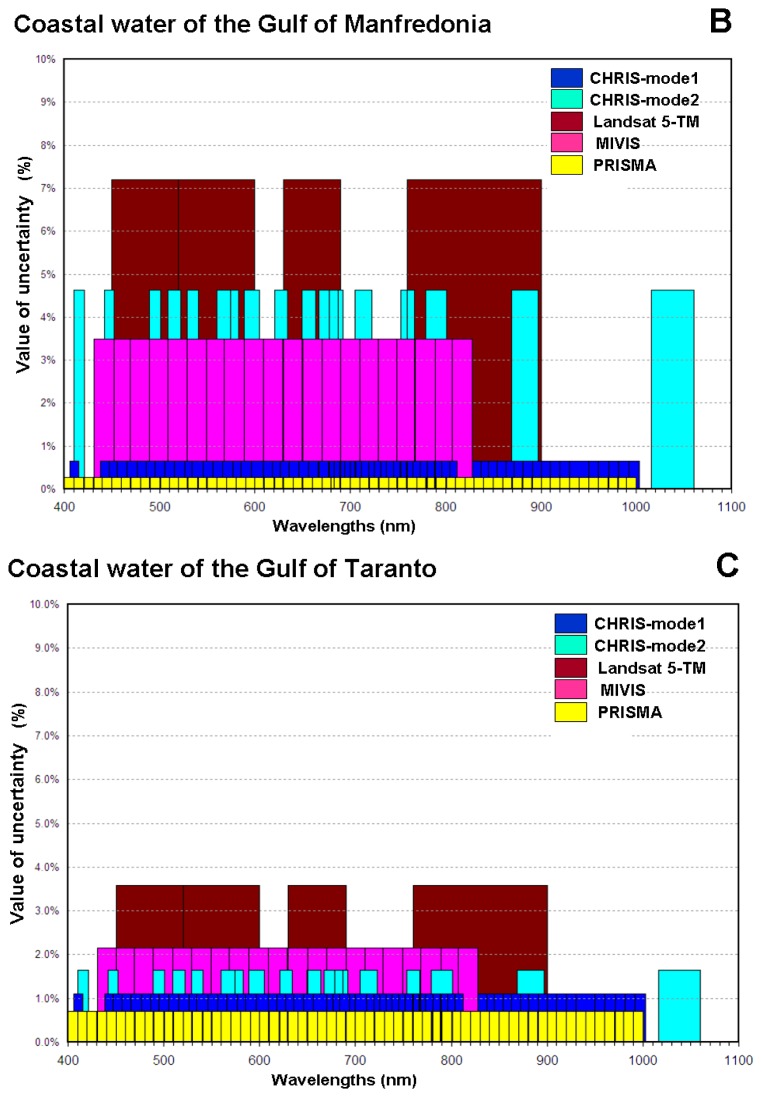
The values of the uncertainty with respect to the spectral characteristics of the data: (**A**) the values of the uncertainty with which these data characterize the coastal waters of the area close to the Lagoon of Lesina; (**B**) the values of the uncertainty, with which these data characterize the coastal waters of the Gulf of Manfredonia; (**C**) the values of the uncertainty, with which these data characterize the coastal waters of the Gulf of Taranto.

In conclusion, the evaluation of these accuracies, with which these data characterize these coastal waters, show that these value are directly proportional to the number of the bands, consequently are inversely proportional to the FWHM of these data. Although these values confirm a logical conclusion, however the must important result is the assessment of the accuracy value. Figure 3 shows the values of the uncertainty, with which these data characterize each coastal waters, with respect to the spectral characteristics of the data.

**Figure 4.**
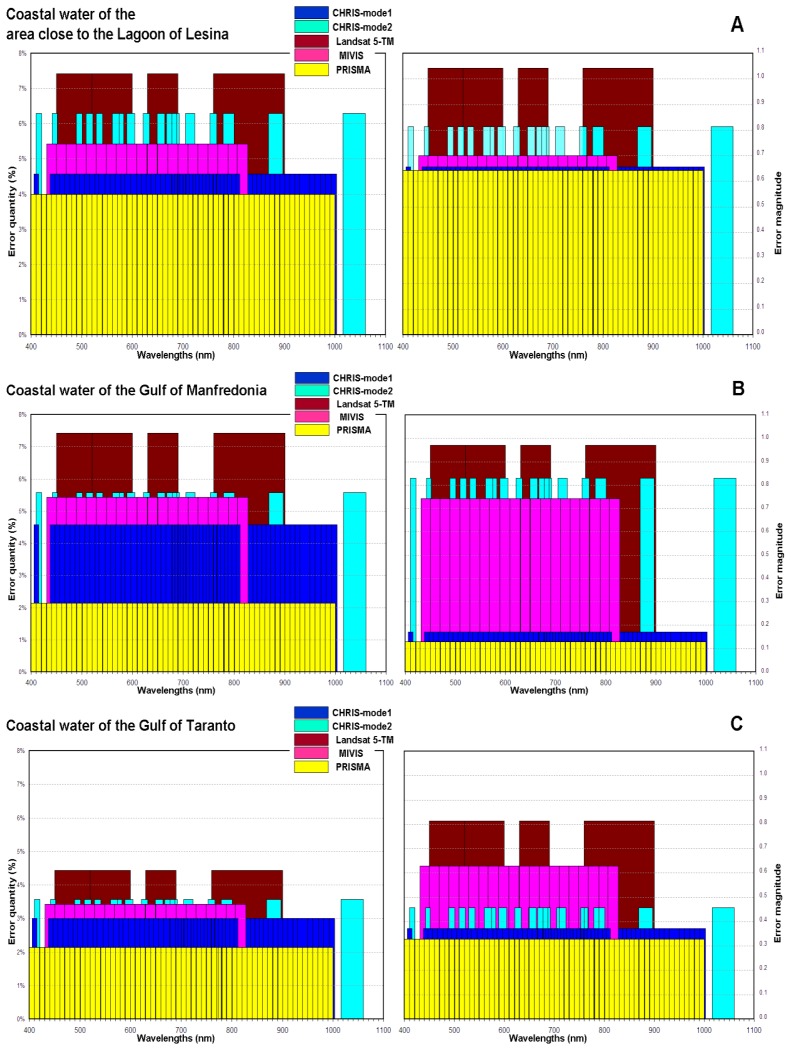
The error quantities (**left**) and the error magnitudes (**right**) with respect to the spectral characteristics of these data: (**A**) error quantities and error magnitudes in characterizing the coastal waters of the area close to the Lagoon of Lesina; (**B**) error quantities and error magnitudes in characterizing the coastal waters of the Gulf of Manfredonia; (**C**) error quantities and error magnitudes in characterizing the coastal waters of the Gulf of Taranto.

Figure 4 shows the error quantities and error magnitudes in characterizing each coastal water with respect to the spectral characteristics of these data.

The values of the uncertainty cover the range from 0.28% to 7.75% as a function of the data (see Table 5). Actually, the uncertainty values with which the PRISMA and the CHRIS mode 1 data characterize these coastal waters, are comparable with the values which are related to the atmospheric correction [36]. While the uncertainty values with which the CHRIS mode 2 and the MIVIS data characterize these coastal waters, are greater than the values related to the atmospheric correction [36], and are lower than the values related to the calibration [32,34]. Lastly the uncertainty values with which the Landasat5-TM data characterize these coastal waters are greater than the values related to the atmospheric correction [36], and are comparable with the values related to the calibration [32,34].

The coastal waters of the area close to the Lagoon of Lesina show the highest uncertainty values with respect to the other coastal waters, while the coastal waters of the Gulf of Taranto show the lowest uncertainty values with respect to the other coastal waters (see Table 5).

As a rule, the values of the uncertainties are inversely proportional to the number of the bands (see Table 5 and Figure 3) and the error quantities and the error magnitudes are directly proportional to the number of the bands (see Table 5 and Figure 4). Only the value of the uncertainty with which the CHRIS mode 2 data (*i.e.*, 18 bands) characterize the coastal waters of the Gulf of Taranto, is lower than the value of the uncertainty with which the MIVIS data (*i.e.*, 20 bands) characterize the same coastal waters. This trend is due to the error magnitude of the CHRIS mode 2 data, which is lower than the value of the MIVIS data (*i.e.*, 0.46 and 0.63 respectively, see Table 5).

As a rule, the distributions of the uncertainty values of each coastal area (see Table 5 and Figure 3) show different characteristics from the others. Only the distribution of the uncertainty values, with which the CHRIS mode 2, the Landasat5-TM and the MIVIS data characterize the coastal waters of the area close to the Lagoon of Lesina (see Figure 3A), shows a slight difference with respect to the distribution of the uncertainty values with which the CHRIS mode 2, the Landasat5-TM and the MIVIS data characterize the coastal waters of the Gulf of Manfredonia (see Figure 3B). These different distributions highlight that the uncertainty values, error quantities and error magnitudes are closely related to the spectral characteristics of the studied area. As a matter of fact, each reflectance of these studied areas shows different spectral characteristics from the other areas.

In conclusion, the purpose of this methodology is to identify the best remote data that improves the characterization of a surface, evaluating the number of bands in the spectral range. In this application, for example, the choice of the CHRIS mode 2 satellite configuration with respect to the choice of the CHRIS mode 1 satellite configuration decreases the accuracy value (or increases the uncertainty value), with which these data characterize the coastal waters of the area close to the Lagoon of Lesina, the Gulf of Manfredonia and the Gulf of Taranto. As a matter of fact, the uncertainty values of the CHRIS mode 2 data, with which this data characterize the coastal waters of the area close to the Lagoon of Lesina, the Gulf of Manfredonia and the Gulf of Taranto, are equal to 5.12%, 4.62% and 1.63%, respectively; while the uncertainty values of the CHRIS mode 1 data are equal to 3.00%, 0.66% and 1.11%, respectively. A deliberate choice is very important especially for characterizing the coastal waters of the Gulf of Manfredonia; in this coastal area the difference between the value of the uncertainty of the CHRIS mode 2 data and the value of the uncertainty of the CHRIS mode 1 data is equal to 3.96%.

In additional, this methodology can explore the advantage to employ the integrated approach [56] to characterize a specific area. In other words, this methodology can identify the best set of remote data that improves the characterization of a surface. In this application for example, the Gulf of Taranto, with respect to the other coastal area, can mainly take advantage of the integrated approach to characterize their coastal waters. As a matter of fact, the values of the uncertainty, with which the CHRIS mode 1 and mode 2, the Landsat5-TM, the MIVIS and the PRISMA data characterize the coastal waters of the Gulf of Taranto, are the lowest with respect to the values of the other coastal area.

## 5. Conclusions

The recent increased availability of the remote sensors allows one to choose between different remote datasets. This paper proposes a methodology to guide the selection of the best remote dataset which improves the spectral characterization of the studied surfaces. Therefore, this methodology leads us to explore a spectral characteristic of the remote data: FWHM. This methodology evaluates the accuracy value with which remote data characterize a specific surface as a function of FWHM evaluating the number of the bands in the specific spectral range. The proposed methodology comprises three steps:

The first step is performed to provide an accurate dataset. This dataset fully describes the “natural” combination and the “natural” variability of the reflectances of the studied surface. In addition, this dataset also describes the “natural” variability of each reflectance. This particular attention, used to create the dataset, is needed to better simulate the remote data. This simulation avoids the atmospheric effects and the noise of the remote sensor, but highlights only this spectral characteristic of the sensor.

The second step is performed to evaluate the capability of the remote simulated data in characterizing a specific surface. This evaluation is based on the ability of the remote data in measuring the spectral similarity between one spectrum and another of this surface. The measurements of the spectral similarity are obtained by using the principal supervised classifiers. Each classifier uses a specific algorithm to measure the spectral similarity between two spectra. This step takes into consideration the overall results of all principal supervised classifiers, because the overall results completely explain the spectral similarity measurements and fully illustrate the spectral characteristics of the surface. Therefore, the overall results fully describe the capability of remote data in characterizing the reflectance of a specific surface. Furthermore, the evaluation of the overall results provides a more versatile methodology, which can characterize every variability of each surface and every combination of reflectances.

The third step assesses the accuracy value with which remote data characterize a specific surface, as a function of FWHM. This step is based on the assumption that *in situ* hyperspectral data more accurately characterize the reflectances of the surfaces with respect to the other remote data. Therefore, the spectral similarity measurements, which are obtained by *in situ* hyperspectral data, more accurately characterize the reflectances of the surfaces with respect to the measurements which were obtained by the other remote data. In conclusion, their spectral similarity measurements are considered to be the “real” measurements. Thus, the assessment of the accuracy is performed by comparing the spectral similarity measurements of the remote data with respect to the “real” spectral similarity measurements. These “real” spectral similarity measurements are obtained from a corresponding synthetic data which is obtained from the *in situ* hyperspectral reflectances, which are resized with the same spectral range of remote data. In other words, these data are characterized by the same spectral range of the remote data, but its FWHM is equal to the FWHM of the *in situ* hyperspectral measurements.

The accuracy value with which remote data characterize a specific surface as a function of FWHM is provided by the quantity and by the magnitude of the differences. These differences between the spectral similarity measurements of the remote data and the “real” spectral similarity measurements are considered “characterization errors”. The quantity and the magnitude of these errors are obtained by the overall spectral similarity measurements which were obtained by all principal supervised classifiers, in order to fully optimise the description and the characterization of a specific surface. Actually, the error quantity is determined by the mean percentage of the quantity of all errors (*i.e.*, the quantity of all differences with respect to the total interactions among the spectra) which are obtained by all performed classifiers. The error magnitude is determined by the mean magnitude of all errors (*i.e.*, the magnitude of all errors with respect to the total interactions among the spectra and the period which is used to normalized the Spectral Similarity Matrices) which are obtained by all performed classifiers. Therefore the error quantity multiplied by the error magnitude provides the value of the uncertainty, with which remote data characterize a specific surface as a function of FWHM.

This methodology was applied to evaluate the accuracy values with which the CHRIS mode 1 and mode 2, the Landsat5-TM, the MIVIS and the PRISMA data characterize the coastal waters of the area close to the Lagoon of Lesina, the Gulf of Manfredonia and the Gulf of Taranto.

The evaluation of the accuracy values, with which these data characterize these coastal waters, show that these value are inversely proportional to the FWHM of the analyzed data. Although these values confirm a logical conclusion, however the must important result is the assessment of the accuracy value. To sum up, the values of the uncertainty, with which these data characterize the coastal waters of the area close to the Lagoon of Lesina, the Gulf of Manfredonia and the Gulf of Taranto, as a function of FWHM are:

- 3.00%, 0.66% and 1.11% respectively, extracted by the CHRIS mode 1 data;
- 5.12%, 4.62% and 1.63% respectively, extracted by the CHRIS mode 2 data;
- 7.75%, 7.22% and 3.61% respectively, extracted by the Landsat5-TM data;
- 3.80%, 3.50% and 2.16% respectively, extracted by the MIVIS data;
- 2.56%, 0.28% and 0.70% respectively, extracted by the PRISMA data.

These results highlight that the application of this methodology is very important to indentify the best remote data which will improve the spectral characterization of the studied surfaces. In additional, this methodology can explore the advantage to employ the integrated approach to characterize a specific area. As a matter of fact, the identification of the best remote dataset, as a function of the FWHM, for characterizing of the studied surfaces, can meaningfully improve the quality of the results.

## Figures and Tables

**Table 1.** The products and the values, which are obtained by this methodology, are shown. Each row describes a step.

**Step**	**Product**	**Value**		
**1^st^**	**Accurate dataset**	“Natural” combination and “natural” variability of the specific surface		All reflectances which characterize the specific surface
		“Natural” variability of each reflectance		Mean and standard deviation values of the measurements
**2^nd^**	**Capability of remote data in characterizing the reflectance of a specific surface**	Capability		Mean value, obtained by all classifiers, of the variety of the spectral similarity measurements divided by the number of the reflectances, which characterize a specific surface
**3^rd^**	**Accuracy, with which remote data characterize a specific surface, as a function of FWHM**.	Uncertainty	Error quantity	Mean percentage of the quantity of all errors (*i.e.*, the quantity of all differences with respect to the total interactions among the spectra) which are obtained by all performed classifiers
			Error magnitude	Mean magnitude of all errors (*i.e.*, the magnitude of errors with respect to the total interactions among the spectra and the period which is used to normalized the Spectral Similarity Matrices) which are obtained by all performed classifiers

**Table 2.** The spectral characteristics of the remote data and the corresponding synthetic data are shown. Each row describes the two elements of each pair. The first group of six columns, in grey, summarizes the spectral characteristics of the remote data for each pair of data. The second group of six columns, in yellow, highlights the spectral characteristics of the synthetic data corresponding to the remote data for each pair of data. The spectral ranges of the CHRIS mode 1, the CHRIS mode 2, the Landasat5 TM and their corresponding synthetic data are not continuously covered.

**Data pair**	**Remote Data**						**Synthetic Data**					
	
	**Sensor**	**Spectral Range**			**Spectral Resolution**	**Spectral Bands**	**Sensor**	**Spectral Range**			**Spectral Resolution**	**Spectral Bands**
	
		**Lower Limit (nm)**	**Upper Limit (nm)**	**Continuous**	**FWHM (nm)**	**Number of Channels**		**Lower Limit (nm)**	**Upper Limit (nm)**	**Continuous**	**FWHM (nm)**	**Number of Channels**
**1st**	CHRIS mode 1	406	1,003	no	Variable from 6 to 20	62	ASD	406	1,003	no	1	561
**2nd**	CHRIS mode 2	410	1,060	no	Variable from 5.7 to 43.7	18	ASD	410	1,060	no	1	291
**3rd**	Landasat5 TM	450	900	no	Variable from 70 to 140	4	ASD	450	900	no	1	353
**4th**	MIVIS	431	828	yes	Variable from 18.1 to 21.1	20	ASD	431	828	yes	1	398
**5th**	PRISMA	400	1,000	yes	10	60	ASD	400	1,000	yes	1	601

**Table 3.** The seven Spectral Similarity Matrices of the PRISMA data, obtained by the seven supervised classifiers which were performed on the coastal waters of the area close to Lagoon of Lesina are shown. The names of the algorithms are shown in the first row of the first column of each Spectral Similarity Matrix, highlighted in yellow. Each Spectral Similarity Matrix is symmetrical with respect to the main diagonal, highlighted in grey. The first row and the first column of each Spectral Similarity Matrix show the station names (see Figure 1a).

**BE**		**Station names**						**Pa**		**Station names**					
		**LES11**	**LES10**	**LES8**	**LES6**	**LES4**	**LES2**			**LES11**	**LES10**	**LES8**	**LES6**	**LES4**	**LES2**
**Station names**	**LES11**	99.70	99.00	97.70	98.00	99.00	98.00	**Station names**	**LES11**	601	317	310	297	296	271
	**LES10**	99.00	99.70	98.00	99.00	99.00	99.00		**LES10**	317	601	421	401	421	397
	**LES8**	97.70	98.00	99.30	99.00	98.00	98.00		**LES8**	310	421	601	403	412	217
	**LES6**	98.00	99.00	99.00	99.70	98.00	99.00		**LES6**	297	401	403	601	587	570
	**LES4**	99.00	99.00	98.00	98.00	99.70	99.00		**LES4**	296	421	412	587	601	124
	**LES2**	98.00	99.00	98.00	99.00	99.00	99.30		**LES2**	271	397	217	570	124	601
**ML**		**Station names**						**SAM**		**Station names**					
		**LES11**	**LES10**	**LES8**	**LES6**	**LES4**	**LES2**			**LES11**	**LES10**	**LES8**	**LES6**	**LES4**	**LES2**

**Station names**	**LES11**	0.00	0.00	0.00	0.00	0.00	0.00	**Station names**	**LES11**	0.0090	0.0640	0.1010	0.0630	0.0380	0.0580
	**LES10**	0.00	0.00	0.00	0.00	0.00	0.00		**LES10**	0.0640	0.0030	0.1240	0.0590	0.0330	0.0560
	**LES8**	0.00	0.00	0.00	0.00	0.00	0.00		**LES8**	0.1010	0.1240	0.0050	0.0760	0.1210	0.0800
	**LES6**	0.00	0.00	0.00	0.00	0.00	0.00		**LES6**	0.0630	0.0590	0.0760	0.0170	0.0600	0.0320
	**LES4**	0.00	0.00	0.00	0.00	0.00	0.00		**LES4**	0.0380	0.0330	0.1210	0.0600	0.0010	0.0580
	**LES2**	0.00	0.00	0.00	0.00	0.00	0.00		**LES2**	0.0580	0.0560	0.0800	0.0320	0.0580	0.0210
**MaD**		**Station names**						**SID**		**Station names**					
		**LES11**	**LES10**	**LES8**	**LES6**	**LES4**	**LES2**			**LES11**	**LES10**	**LES8**	**LES6**	**LES4**	**LES2**

**Station names**	**LES11**	3.50	4.75	5.00	4.90	4.80	4.80	**Station names**	**LES11**	0.0000	0.0070	0.0210	0.0220	0.0090	0.0160
	**LES10**	4.75	3.50	4.90	5.10	4.90	4.90		**LES10**	0.0070	0.0010	0.0120	0.0090	0.0040	0.0065
	**LES8**	5.00	4.90	3.50	4.90	4.75	5.30		**LES8**	0.0210	0.0120	0.0010	0.0060	0.0150	0.0070
	**LES6**	4.90	5.10	4.90	3.50	4.90	4.75		**LES6**	0.0220	0.0090	0.0060	0.0000	0.0070	0.0020
	**LES4**	4.80	4.90	4.75	4.90	3.50	4.90		**LES4**	0.0090	0.0040	0.0150	0.0070	0.0000	0.0038
	**LES2**	4.80	4.90	5.30	4.75	4.90	3.50		**LES2**	0.0160	0.0065	0.0070	0.0020	0.0038	0.0010
**MiD**		**Station names**													
		**LES11**	**LES10**	**LES8**	**LES6**	**LES4**	**LES2**								

**Station names**	**LES11**	0.0130	0.0410	0.1570	0.1640	0.1580	0.1850								
	**LES10**	0.0410	0.0200	0.1450	0.1470	0.1400	0.1670								
	**LES8**	0.1570	0.1450	0.0130	0.0390	0.0510	0.0570								
	**LES6**	0.1640	0.1470	0.0390	0.0190	0.0260	0.0390								
	**LES4**	0.1580	0.1400	0.0510	0.0260	0.0080	0.0450								
	**LES2**	0.1850	0.1670	0.0570	0.0390	0.0450	0.0310								

**Table 4.** The varieties of the spectral similarity measurements, extracted by each classifier for each site, are shown. These varieties were obtained on the CHRIS mode 1 and mode 2, the Landsat5-TM, the MIVIS, the PRISMA and the ASD, resized from 400 to 1,000 nm, data. In the last two columns, the total variety of the spectral similarity measurements and the capability are shown.

**Coastal waters of**	**Sensor**	**Variety of Each Classifier**							**Total Variety**	**Capability**
		**BE**	**ML**	**MaD**	**MiD**	**Pa**	**SAM**	**SID**		
**The area close to the Lagoon of Lesina**	**CHRIS mode 1**	4	1	7	16	12	17	13	70	**1.67**
	**CHRIS mode 2**	3	1	6	15	11	16	11	63	**1.50**
	**Landasat5 TM**	2	1	2	10	10	10	8	43	**1.02**
	**MIVIS**	4	1	5	14	10	15	11	60	**1.43**
	**PRISMA**	5	1	7	16	12	17	13	71	**1.69**
	**ASD (400–1,000 nm)**	7	1	9	17	16	20	14	84	**2.00**
**The Gulf of Manfredonia**	**CHRIS mode 1**	16	1	2	88	66	88	80	341	**1.35**
	**CHRIS mode 2**	14	1	2	89	55	88	79	328	**1.30**
	**Landasat5 TM**	4	1	2	88	13	81	76	265	**1.05**
	**MIVIS**	17	1	2	90	55	87	76	328	**1.30**
	**PRISMA**	17	1	2	93	64	88	79	344	**1.37**
	**ASD (400–1,000 nm)**	23	1	42	93	67	88	79	393	**1.56**
**The Gulf of Taranto**	**CHRIS mode 1**	6	1	2	77	77	77	66	306	**2.08**
	**CHRIS mode 2**	6	1	2	80	57	72	62	280	**1.90**
	**Landasat5 TM**	2	1	2	76	13	69	57	220	**1.50**
	**MIVIS**	3	1	2	75	61	77	60	279	**1.90**
	**PRISMA**	7	1	2	74	77	78	68	307	**2.09**
	**ASD (400–1,000 nm)**	13	1	30	76	78	80	68	346	**2.35**

**Table 5.** The total number of the all errors, the error quantity, the total magnitude of all errors, the error magnitude and the value of the uncertainty (highlighted with darker color), which were obtained by the five data pairs for the three coastal sites, are shown.

	**Data Pair**				

	**CHRIS Mode 1 Data and Its Corresponding Synthetic Data**	**CHRIS Mode 2 Data and Its Corresponding Synthetic Data**	**Landsat5-TM Data and Its Corresponding Synthetic data**	**MIVIS Data and Its Corresponding Synthetic Data**	**PRISMA Data and Its Corresponding Synthetic Data**
Coastal Area Close to the Lagoon of Lesina					
Total number of all errors	47	64	77	57	41
Error quantity	4.57%	6.29%	7.43%	5.43%	4.00%
Total magnitude of all errors	681	845	1,073	725	656
Error magnitude	0.66	0.81	1.04	0.70	0.64
Uncertainty	3.00%	5.12%	7.75%	3.80%	2.56%
Gulf of Manfredonia					
Total number of all errors	1,265	1,826	2,429	1,518	704
Error quantity	3.86%	5.57%	7.43%	4.71%	2.14%
Total magnitude of all errors	5,476	26,847	31,676	24,472	4,399
Error magnitude	0.17	0.83	0.97	0.74	0.13
Uncertainty	0.66%	4.62%	7.22%	3.50%	0.28%
Gulf of Taranto					
Total number of all errors	642	762	1,051	713	390
Error quantity	3.00%	3.57%	4.43%	3.43%	2.14%
Total magnitude of all errors	4,258	5,160	9,214	7,068	3,755
Error magnitude	0.37	0.46	0.81	0.63	0.33
Uncertainty	1.11%	1.63%	3.61%	2.16%	0.70%
